# A Comparative Study of the Outcomes of Surgically Versus Non‐Surgically Treated Spinal Disease in Patients With Multiple Myeloma: Northern Ireland's Experience

**DOI:** 10.1002/jha2.70071

**Published:** 2025-06-01

**Authors:** Amber McCalmont, Nagy Darwish, David Donaldson, Eiman Abdel Meguid

**Affiliations:** ^1^ Queen's University Belfast School of Medicine Dentistry and Biomedical Sciences Belfast UK; ^2^ Royal Victoria Hospital Trauma and Orthopaedics Belfast UK; ^3^ Belfast City Hospital Health and Social Services Trust Haematology, Belfast City Hospital Belfast UK

**Keywords:** multiple myeloma, spinal disease, spinal surgery

## Abstract

**Background:**

Multiple myeloma is a haematological malignancy which is characterised by the proliferation of cancerous plasma cells in the bone marrow. The role of spinal surgery in the management of myeloma is debatable. Therefore, this retrospective study aimed to compare the outcomes of treatment of patients with myeloma whose spinal disease was managed surgically and/or non‐surgically.

**Methods:**

A total of 159 patients were reviewed retrospectively. To compare the outcomes of treatment in both cohorts, three outcome measures were selected, including back‐pain reduction, neurological status, and survival duration.  In addition, the extent and distribution of vertebral disease was assessed using MRI Whole Spine reports.

**Results:**

There was no significant difference in the percentage of patients in each cohort reporting back‐pain pretreatment and at the two follow‐up periods (*p* > 0.05). Regarding neurological status, 23% of the surgical cohort improved, 53% remained stable and 7% deteriorated. In comparison, the non‐surgical cohort displayed no significant changes in neurological status post‐treatment. The mean duration of survival was significantly longer in the cohort who received surgery (77 vs. 24 months, *p* = 0.014). However, the mean age of diagnosis was significantly lower in this cohort (59 vs. 71 years, *p* < 0.001). T12 was the most commonly diseased vertebral level across both cohorts. At the time of diagnosis, the average number of diseased vertebrae per patient was 3.5 in the surgical cohort and 3.6 in the non‐surgical cohort.

**Conclusion:**

This comparative study has shown that back pain alone should not be an indication for spinal surgery. However, surgical intervention may successfully prevent neurological deterioration. Although surgical intervention is associated with prolonged survival, this may be confounded by demographic variables, such as age. Importantly, most patients displayed multi‐level disease at the time of diagnosis.

**Trial Registration:**

The authors have confirmed clinical trial registration is not needed for this submission.

## 1 Introduction

Multiple myeloma is a haematological malignancy which is characterised by the proliferation of cancerous plasma cells in the bone marrow [1]. Plasma cells are activated B lymphocytes which produce immunoglobulins [2]. The proliferation of malignant plasma cells within the bone marrow disrupts skeletal homeostasis. This results in increased bone resorption through heightened osteoclast activity and inhibited osteoblast activity. The progressive destruction of bone which occurs during the disease process may lead to the formation of pathological fractures. In multiple myeloma, the vertebral column is the most common site for pathological fractures to occur. Lytic lesions most commonly affect the vertebral bodies where the bone marrow concentration is the greatest. However, other regions of the vertebrae may also be affected [3]. In patients with myelomatous spinal involvement, lower back pain is the most common presenting symptom [4]. This correlates with the fact that the marrow quantity is the greatest in the lumbar region where the vertebral bodies are the largest. Furthermore, it is estimated that around 60% of individuals with multiple myeloma will already have bony lesions in the spine at the time of diagnosis, although they may be asymptomatic [1].

There is debate in the medical community as to whether there is a role for spinal surgery in the management of spinal disease in multiple myeloma [3]. The long‐term efficacy of implemented surgical devices may be affected by the extensive destruction of bone which occurs during the disease process. As the pattern of spinal involvement is typically diffuse, adverse outcomes may arise when stabilisation of the spine is attempted by the surgeon [5]. Moreover, as patients with myeloma may be immunocompromised, they are at an increased risk of developing postoperative infections [2].

Therefore, this study aimed to determine whether there is a role for spinal surgery in the management of patients with myelomatous spinal pathology. The aim was to compare the outcomes of treatment when managed surgically versus non‐surgically. The chosen outcome measures were pain reduction, neurological function and survival duration post‐treatment. An additional aim of the study was to assess the extent and distribution of vertebral disease.

## 2 Methods

A total of 159 patients who received treatment for multiple myeloma between October 2010 and January 2023 in Northern Ireland were reviewed retrospectively. To compare patients' outcomes when treated surgically versus non‐surgically, two cohorts were required. The first cohort contains patients with multiple myeloma who underwent spinal surgery. To form a comparative group, patients with myeloma affecting the spine who did not receive surgery were selected. Thus, the two cohorts which were compared with each other are the surgical cohort and the non‐surgical cohort.

**FIGURE 1:**
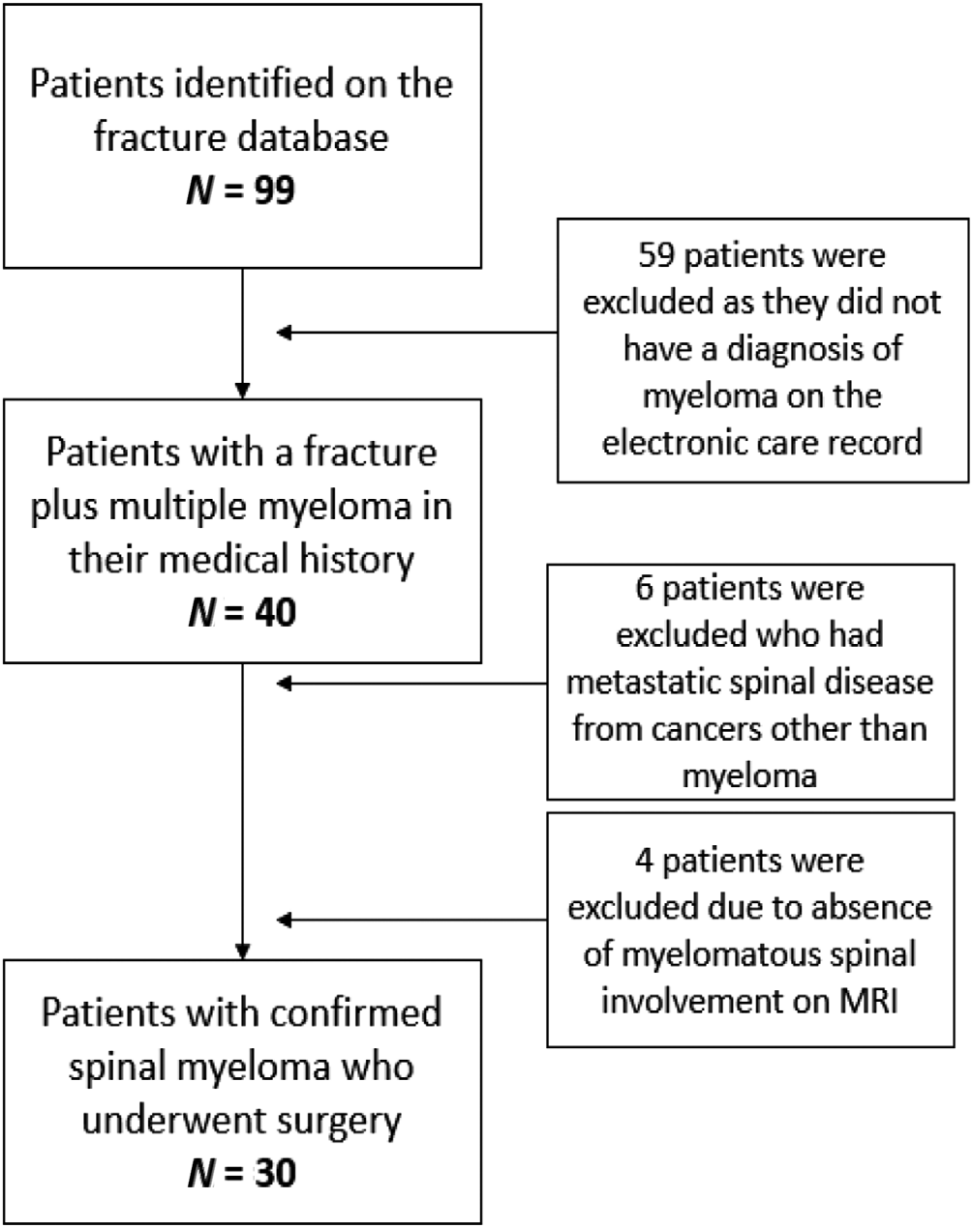
A flowchart to demonstrate how the surgical cohort was formed after inclusion and exclusion criteria were applied.

**FIGURE 2:**
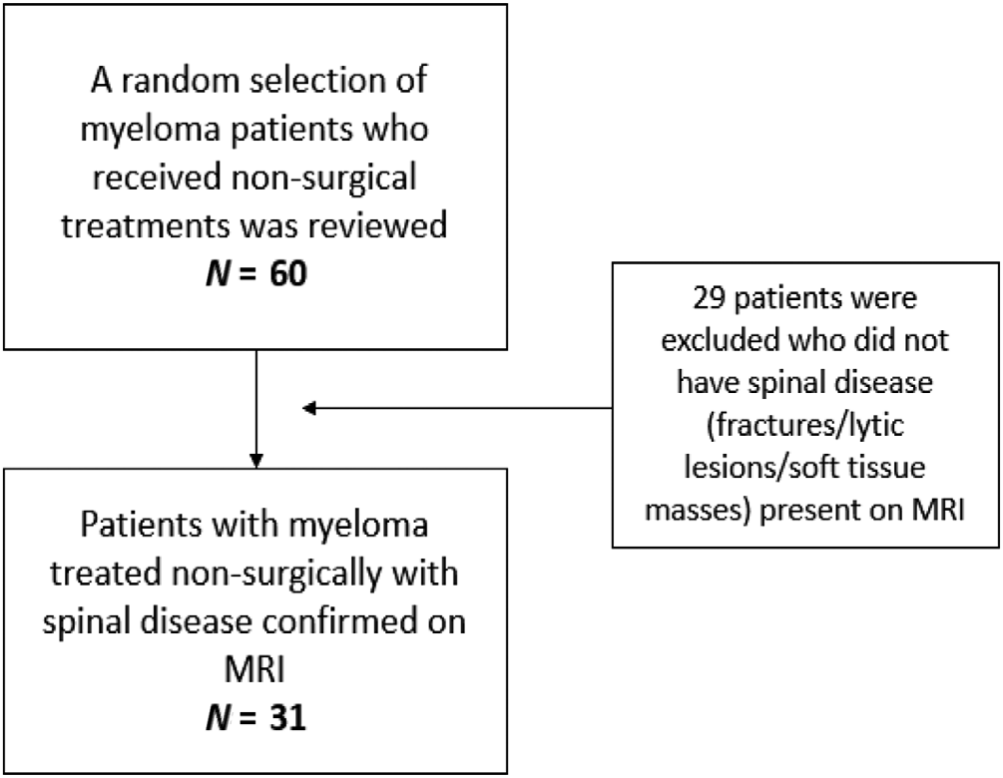
A flowchart to display the application of inclusion and exclusion criteria to the non‐surgical cohort of patients.

The patients in the surgical cohort were recruited using data from the Fracture Outcomes Research Database (Ford), as shown in Figure 1. To form the non‐surgical cohort, a random sample of 60 patients with multiple myeloma was reviewed (Figure 2). The following inclusion and exclusion criteria were applied:

Inclusion Criteria:

- A confirmed diagnosis of multiple myeloma.
- The receipt of treatment for multiple myeloma between October 2010 and January 2023.
- Three and six months' follow‐up information available on the electronic care record.
- ‘MRI Whole Spine’ obtained and interpreted with evidence of myelomatous spinal pathology.
- Spinal surgery performed in the Royal Victoria Hospital (for the surgical cohort only).

Exclusion Criteria:

- No evidence of spinal disease on MRI, such as fractures, vertebral wedging or lytic lesions.
- The presence of metastatic spinal disease from a cancer other than myeloma.
- No attendance at 3 or 6 months' follow‐up appointments.

The outcome measures which were evaluated in this study were pain reduction, neurological function and survival duration post‐treatment. The vertebral levels affected during the disease process have also been evaluated. To assess the symptoms experienced by patients in each cohort, clinical documents available on patients' electronic care record (ECR) were reviewed. Pain levels were not recorded quantitively in any of the clinical documents available for both cohorts. Therefore, pain was noted as being present, absent, improved or worsened.

Patients’ neurological function was classified using the Frankel grading system pretreatment and at 6 months’ follow‐up (Table 1). The Frankel grade is a widely used scale in orthopaedics [6].

**TABLE 1:** The Frankel Grade classification for assessing spinal cord function below the level of spinal cord injury [6].

Grade	Description of clinical signs
A	No motor or sensory function
B	No motor function; Some sensory function
C	Non‐functional motor function, that is, severe weakness, ± sensory function
D	Voluntary motor function preserved for useful functioning
E	Normal sensation and motor control below level of lesion

The length of time between diagnosis and treatment, treatment and death, and diagnosis and death were calculated for each patient. The mean duration of survival in each cohort was subsequently calculated and recorded in months. The mean survival durations were compared to evaluate whether surgical intervention was associated with prolonged or reduced survival.

An ‘MRI Whole Spine’ was performed prior to treatment to assess the extent of spinal pathology. The MRI reports for each patient were subsequently reviewed to assess the distribution of vertebral pathology.

## 3 Results

The surgical cohort consisted of 30 patients, comprising 22 males and 8 females with a mean age of 59 at the time of diagnosis (range: 33–83). The surgical procedures performed were as follows: 19 patients underwent posterior decompression and stabilisation; 10 patients underwent vertebroplasty/kyphoplasty; and 1 patient had a combined anterior corpectomy with posterior stabilisation.

The non‐surgical cohort consisted of 31 patients with multiple myeloma who did not undergo spinal surgery. This comprised 20 males and 11 females. The average age at the time of diagnosis was 71 (range: 33–83). Five patients (16%) received radiotherapy to the spine during the follow‐up period.

**FIGURE 3:**
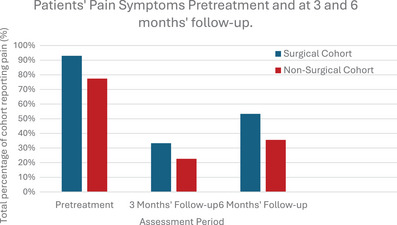
A bar chart to display the percentage of patients in each cohort whose pain persisted or did not improve at follow‐up.

Throughout the study period, a higher percentage of the surgical group reported pain at all stages compared to the non‐surgical group. The percentages include the total number of patients whose pain did not improve or was present at follow‐up period (Figure 3). Pretreatment, 93.0% of the surgical patients reported back pain symptoms compared to 77.4% in the non‐surgical group. However, the percentage reduction in pain at the 3‐month follow‐up period was greater in the surgical group (60% reduction vs. 54.8%). Between the 3 and 6 months' follow‐up period, the percentage of patients whose pain returned was 20.3% in the surgical cohort and only 12.9% in the non‐surgical cohort.

The most common pretreatment symptom in both cohorts was lower back pain which was either acute or chronic in character. Some patients described pain in the cervical and thoracic regions. However, there was no significant difference in back pain reduction between the two groups after statistical analyses were performed using chi‐squared tests (*p* = 0.157).

**FIGURE 4:**
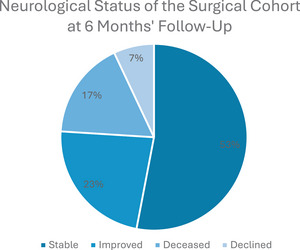
A pie chart to display the change in neurological status of the patients in the surgical cohort 6 months postoperatively.

In the surgical group, 18 patients presented with Frankel Grade E, 4 patients were Frankel Grade D, 5 were Frankel Grade C and 2 patients were Frankel Grade A. Postoperatively, 16 patients' Frankel Grade remained stable, 7 patients' neurological status improved Frankel grades and 2 patients' Frankel Grade deteriorated. The changes in patients' neurological status from the preoperative period compared to the 6‐month follow‐up period are summarised in Figure 4.

In comparison, the non‐surgical group displayed no significant changes in neurological status post‐treatment. One patient from the non‐surgical cohort had leg weakness prior to treatment which persisted 6 months post‐treatment. Another patient reported tingling in the fingers in conjunction with lower cervical spine pathology affecting nerve roots C6, C7 and C8. Their neurological deficits were present pre‐ and post‐treatment.

The mean age of diagnosis in the surgical group was 59, compared to 71 in the non‐surgical group. The 12‐year difference in the mean age of diagnosis was significant (*p* < 0.001). Furthermore, the mean duration of survival was significantly longer in the surgical cohort (*p* = 0.014). The demographic and survival details are summarised in Table 2. Patients' stage of disease at the time of diagnosis was calculated using the Revised Multiple Myeloma International Staging System [7].

**TABLE 2:** A table summary of the demographic details and mean survival outcomes in the surgical and non‐surgical cohorts.

Cohort	Surgical	Non‐surgical
Sex	22 males and 8 females	20 males and 11 females
Mean age of diagnosis	59 years (Range: 33–83)	71 years (Range: 55–88)
Stage of disease at time of diagnosis	7 Stage 1 20 Stage 2 3 Stage 3	9 Stage 1 17 Stage 2 5 Stage 3
Number in each cohort with high‐risk cytogenetics	1	2
Treatment regimes	Lenalidomide/thalidomide ± dexamethasone ± bortezomib ± cyclophosphamide	Lenalidomide/thalidomide ± dexamethasone/prednisone ± bortezomib ± cyclophosphamide ± melphalan
Mean age of death	65 years	77 years
Number of cohort deceased	19/30 (63.3%)	12/31 (38.7%)
Mean duration between diagnosis and treatment	35 months	3 months
Mean survival from diagnosis	77 months (Range: 4–251 months)	24 months (Range: 1–61 months)
Mean survival postoperative/ post‐ systemic anti‐myeloma survival	40 months	23 months

**FIGURE 5:**
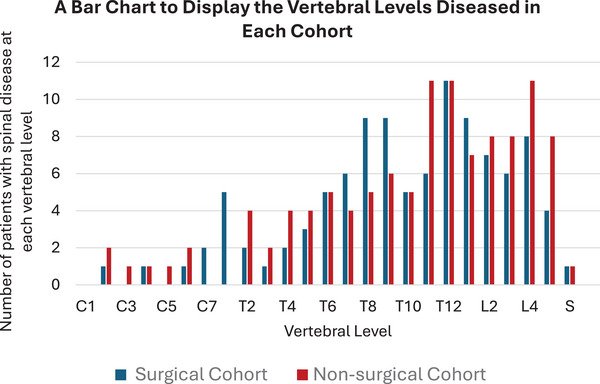
A bar chart to show the vertebrae which were diseased in the surgical and non‐surgical cohorts. The MRI scans performed prior to surgery detailed which levels were affected.

Every patient included in the study displayed evidence of myelomatous spinal pathology. The vertebral levels which were diseased are shown in Figure 5. The pathology present included lytic lesions, pathological fractures, marrow infiltration (diffuse or variegated) and soft tissue masses (e.g. extramedullary plasma cell tumours). The most commonly affected vertebral level in the surgical cohort was T12. A total of 37% of the patients displayed pathology in this vertebra. The second most commonly affected vertebrae were T8, T9 and L1, with 30% of the cohort displaying pathology at these levels. In the non‐surgical cohort, T11, T12 and L4 were the most commonly affected vertebrae (35%). The second most commonly affected levels were L2, L3 and L5 (26%). Therefore, across the two cohorts, the lower thoracic and lumbar vertebrae were affected by myelomatous pathology in most cases. The patients in the surgical cohort had an average of 3.5 vertebral levels affected by myelomatous pathology. In the non‐surgical cohort, the average number of vertebrae diseased per patient was 3.6.

Excluding death, 3 out of the 30 patients in the surgical cohort experienced complications. The postoperative complications were infection (osteomyelitis), incontinence, metalwork collapse and death. Therefore, the postoperative complication rate was 0.1. Further surgery was required to correct collapsed metalwork in 1 out of the 30 surgical patients.

## 4 Discussion

Multiple myeloma is the most common primary malignancy which originates in the spine [8]. Myeloma typically affects the older population, although its aetiology is not yet fully understood [9]. The average age of diagnosis of the patients in the non‐surgical cohort in this study was 71 years. The non‐surgical cohort was representative of the overall population of myeloma patients in Northern Ireland in terms of age, symptoms, treatment and survival [2].

There was a significant difference in the mean age of diagnosis between the surgical and the non‐surgical cohorts (59 vs. 71, *p* < 0.001). Therefore, this study has shown that patients with myeloma who have historically been offered spinal surgery in Northern Ireland are those who have presented at a younger age than average. The younger patients are less likely to have had complex comorbidities, and as such, were more likely to have been deemed medically fit for surgery [10].

In the current study, the median survival in the surgical cohort was 58 months (mean = 77 months; range: 4–251 months). In the non‐surgical cohort, the median survival was 27 months (mean = 24 months; range: 1–61 months). Although the mean duration of survival was significantly longer in the surgical cohort (*p* = 0.014), multiple myeloma is currently incurable and surgery cannot prevent disease progression. Therefore, the difference in survival may be related to the fact that the surgical patients were diagnosed at a younger age, presumably with less comorbidities and a higher baseline fitness prior to treatment. The conservative treatments that both cohorts received are more likely to prolong life than surgical intervention alone [11].

The pathophysiology of myeloma is complex, and as such, patients can experience a myriad of symptoms. In the present study, back pain was the most common symptom which patients reported. Similarly, a systematic review of the prevalence of symptoms in myeloma patients found fatigue and back pain to be the most common symptoms [4].

Prior to treatment, back pain was present in 85.2% of the participants. Pain symptoms had decreased notably in both cohorts at the 3‐month follow‐up period. However, the percentage of patients reporting pain had increased at the 6‐month follow‐up period in both cohorts. The increase in pain symptoms may be associated with disease progression over time. Notably, the proportional increase in pain was greater in the surgical cohort compared to the non‐surgical cohort at the 3‐ and 6‐month follow‐up periods. As there was no significant difference in the pain reduction between the two cohorts (*p* > 0.05), pain without neurological impairment should not be an indication for surgery. Patients who are experiencing bone pain may improve with conservative treatments alone. Therefore, pharmacological agents and radiotherapy are important during all stages of disease to slow the progression of bone destruction and to reduce pain levels [12].

Myeloma‐related lesions typically develop in the thoracolumbar spine where the concentration of bone marrow is the greatest [13]. Nervous structures, such as the spinal cord and nerves roots, are subsequently at risk of damage. In 2021, Chen et al. published a study on the management of spinal cord compression in patients with multiple myeloma. It is recommended that known cord compression must be treated urgently to prevent permanent neurological damage [14]. In the absence of neurological sequelae, it is recommended that corticosteroids should be given in combination with radiotherapy [15]. Similarly, this study recommends that patients should be referred to orthopaedics urgently to be considered for surgical intervention if symptoms of neurological impairment are present. This was supported by the fact that 70% of the patients who underwent surgery in Northern Ireland's regional spinal unit had good neurological outcomes at the 6‐month follow‐up period. Outcomes were considered to be good if the patient's neurological status did not deteriorate, or if the Frankel Grade improved.

The patients reviewed in the present study displayed varied pathology in the vertebral column. In the surgical and non‐surgical cohorts, the average number of vertebrae diseased per patient was 3.5 and 3.6, respectively. Notably, Zijlstra et al. (2023) investigated the incidence of vertebral compression fractures in newly diagnosed myeloma patients. Of the 385 patients involved in Zijlstra's study, 47% had at least one vertebral compression fracture at the time of disease onset [16]. The distribution of vertebral disease that was observed in the present study is similar to the distribution documented in the current literature [11]. Overall, the vertebral level which was affected by myeloma in the majority of patients was T12. Fractures in the T12 vertebra are of particular interest to orthopaedic surgeons. T12 is referred to as a ‘junctional’ vertebra, as it is located at the point of transition from the relatively rigid thoracic spine to the more flexible lumbar spine [17].

A large‐scale study of prognostic variables in patients with multiple myeloma found that skeletal related issues, namely pathological fractures, hypercalcaemia of malignancy and spinal cord compression, confer the greatest increase in the relative risk of death compared to other variables. Furthermore, those who experienced a skeletal related issue prior to commencement of bisphosphonate therapy have a 2.5 increased relative risk of death compared to those who start bisphosphonate therapy without a previous skeletal related event [18]. Although the patients in the surgical group had a significantly longer mean survival duration compared to the non‐surgical group (12‐year difference, *p* = 0.014), surgery does not halt the progression of the underlying pathology. Therefore, non‐surgical interventions play an essential role in the management of myeloma to reduce the occurrence of pathological fractures, and to improve survival outcomes. Similarly, an additional study involving 14,013 myeloma patients reported a significantly increased risk of death when pathological fractures were present [19]. Recommendations from the Bone Working Group of the International Myeloma Working Group highlight bisphosphonates and denosumab as key treatments for myeloma‐related bone disease. It is suggested that denosumab may prolong progression‐free survival in newly diagnosed cases. Denosumab may be preferable to bisphosphonate treatment when renal impairment is present and should be considered under close monitoring with a creatinine clearance of < 30 mL/min [20]. Therefore, all newly diagnosed patients should receive the appropriate non‐surgical treatments, including systemic anti‐myeloma therapy, radiotherapy and bisphosphonate medication, as appropriate, to reduce the likelihood of fracture formation and to improve survival outcomes.

A multidisciplinary approach is required to successfully treat and manage patients with multiple myeloma [18]. As the disease is currently incurable, treatment aims to combat disease progression, to preserve quality and length of life and to prevent neurological deterioration [3]. Patients with symptomatic spinal disease must be managed on a case‐by‐case basis. Patients should be referred to orthopaedics if they are experiencing significant pain and/or neurological symptoms. The International Myeloma Working Group reiterated the necessity of orthopaedic consultation in cases of spinal instability with abnormal neurological signs. Patients with symptomatic vertebral compression fractures are recommended to be considered for cement augmentation to prevent potential cord compression. Cement augmentation is deemed to be an excellent procedure for reducing fracture‐related pain, with the additional benefit of not requiring metalwork to be left in the spinal column long‐term, which could pose a serious infection risk to immunocompromised patients. Vertebral kyphoplasty is an alternative management option [21]. The results of this study support these recommendations.

Notably, patients in the surgical cohort experienced postoperative complications at a rate of 0.1. Infection and collapsed metalwork were two of the complications experienced. A study conducted by Milavec et al. (2020) recorded postoperative complications to be present in 21% of myeloma patients who had undergone spinal surgery [8]. This was higher than the complication rate seen in the present study whereby 10% of patients in the surgical cohort experienced a complication in the 6 months following surgery. The incidence of metalwork collapse in the present study was 3.3%. Importantly, the risk of developing complications is higher in patients who receive open decompressive surgery immediately after receiving chemo/radiotherapy [10].

## 5 Conclusion

There was no significant difference in pain reduction in patients managed surgically versus non‐surgically at 3‐ and 6‐month follow‐up. Therefore, patients with painful myelomatous spinal lesions should be managed conservatively. Surgical intervention can successfully prevent the deterioration of neurological function in patients presenting with neurological impairment caused by spinal myeloma. Surgery should not be indicated if neurological symptoms are absent. Furthermore, the vertebral level which was diseased most frequently was T12.

## Author Contributions

Amber McCalmont was the main author of the paper. She contributed to all components of the research and was also responsible for gathering the data which was evaluated in the paper. She performed the statistical analyses and created the relevant figures and tables. Eiman Abdel Meguid was responsible for supervising the project. Her expertise was essential for structuring, reviewing, and editing the paper. Nagy Darwish was an additional supervisor of the project. He was responsible for formulating the idea for the research, as well as reviewing the project throughout its entirety. He contributed significantly to the results and discussion sections. David Donaldson used his clinical expertise to review the paper and contributed especially to the methods and discussion section. He also contributed towards calculating the stages of diagnosis of the patients.

## Ethics Statement

The authors have nothing to report.

## Consent

The authors have nothing to report.

## Conflicts of Interest

The authors declare no conflicts of interest.

## Data Availability

The authors confirm that the data supporting the findings of this study are available within the article (and/or) its Supporting Information.
